# Understanding climate-induced migration in West Africa through the social transformation lens

**DOI:** 10.3389/fsoc.2023.1173395

**Published:** 2023-12-22

**Authors:** Charity Osei-Amponsah, William Quarmine, Andrew Okem

**Affiliations:** Governance and Inclusion Research Group, International Water Management Institute, Accra, Ghana

**Keywords:** climate change, migration, West Africa, climate adaptation, social transformation

## Abstract

The climate crisis has migration implications, and we need to act inclusively and urgently. Climate change impacts people’s decisions to migrate largely through economic, political, technological, demographic, and socio-cultural factors, and their dynamic interlinkages. These complex issues often influence climate risks and vulnerabilities and complicate effective investment and policy actions on migration. However, there is inadequate documentation on how climate change is linked to migration and social transformation. Based on a traditional literature review and inputs from a consultation dialogue, this paper analyzes climate-induced migration in West Africa using a social transformation lens. The paper conceptualizes the climate-induced migrant as an agent of adaptation and describes the complexities of climate vulnerabilities, and its intersection with social transformation in migration decisions. A social transformation conceptual framework is proposed to identify the complexities of climate-induced migration and ensure inclusive strategies are planned, implemented, and sustained. The paper discusses the need for transdisciplinary research approaches to capture various intersections of transforming socio-economic and environmental vulnerabilities across different countries and migratory landscapes. The paper also highlights the critical concern in the region regarding the “trapped population.” It suggests that a social transformation lens is required to unravel the dynamics around vulnerable people unable to migrate because they do not have the resources to migrate or are constrained by cultural issues.

## 1 Introduction

High levels of migration have characterized livelihoods in West Africa for centuries (Mensah-Bonsu, 2003). In 2020, estimated data from the International Migration Stock indicated that there were 7,551,660 million immigrants in the 16 countries of West Africa (UN Population Division, 2020). The reasons for the movement of people include forced displacements and conflicts, transhumance, routine seasonal annual shifts in agriculture, search for employment and better economic opportunities, and impacts of climate change (Adepoju, 2005; Teye and Nikoi, 2022). West Africa is very vulnerable to climate variability and change, largely due to its low adaptive capacity (Turco et al., 2015; Rigaud et al., 2018). The sixth assessment report of IPCC (2022) observed increases in the rate of surface temperature, hot extremes, sea level rise, high-intensity precipitation, as well as frequency and severity of coastal flooding, especially in low-lying areas. These changes, whose effects are exacerbated by increasing poverty rates, gender inequities, population growth and food insecurity, have implications for the agrarian livelihoods of over 80% of the population (Kirwin and Anderson, 2018). The region’s dependence on rainfed agriculture, coupled with high poverty rates, makes it very vulnerable to climate change and variability (IOM, 2020a). The widespread coastal erosion and flooding also pose serious threats to economic opportunities for many fishing communities (Rigaud et al., 2021). The responses include moving out or using other coping strategies to adapt to the changing climate context (Chu and Michael, 2019). Climate change is at least partly responsible for rural–urban migration, as people move in search of alternative livelihoods (Hassan and Tularam, 2018). Henry et al. (2004), found a link between climate (rainfall) and short-distance migration in Burkina Faso. Some household members move to other rural villages with better climatic conditions; for example, young men from the arid regions of northern Ghana migrate to the high-rainfall areas in the south (Abu et al., 2014).

Recently, many people who depend on rainfed agrarian livelihoods consider migration as a climate adaptation response (Teye et al., 2015; Bosetti et al., 2021). Models project that up to 32 million people in West Africa will move within their countries by 2050 due to the impacts of climate change (Rigaud et al., 2021). This analytical estimate was generated from a population gravity model that projects future population distribution for each of the West African countries in the study. The analysis was based on two development scenarios—unequal development (poor development prospects) and moderate development (more equitable future worth). The scenarios were also built on spatial population projections on shared socio-economic pathways of integrated analysis of climate implications of vulnerabilities, adaptation, and mitigation. The region is projected to have over 50 million internal migrants by 2050 at a 2.5°C global warming (CDKN and ACDI, 2022).

Thus, climate-induced migration, i.e., the movement of people driven by sudden or progressive changes in the weather or climate conditions, is gaining increasing interest from researchers, development practitioners and governments (Klepp, 2017; Milán-García et al., 2021; Morrissey, 2021; Thiede et al., 2021; Zickgraf, 2021; Filho et al., 2022). However, there is very little understanding of its dynamics and underlying complexities, hampering the effectiveness of policymakers and investors. This paper analyzes these complexities through a social transformation lens and identifies entry points for research, investments, and policy making.

Migration is both a critical development issue and a social process shaped by socially constructed norms, values, and beliefs (e.g., gender norms and roles). Although migration is one possible response to climate risks, not every household affected by the changing climate will respond with a migration strategy (Black, 2001). Others may be unable to or may choose not to migrate due to poverty, gender, remoteness, ill-health, and age (Kothari, 2003). The decision to migrate, therefore, depends on who is involved, what drives them and how society is transforming. Thus, migration is influenced by a multi-faceted set of factors with social, economic, cultural, political, and demographic consequences (Manou et al., 2017; Zickgraf, 2021; IPCC, 2022) that require in-depth understanding and action. The influencing factors are also dynamic and affect both existing climate risks and vulnerabilities and the coping strategies of households with low adaptive capacity (Rigaud et al., 2021). It is therefore, important to investigate not only climate and migration trends, but also the intersecting dynamic vulnerabilities and adaptive capacities that inform individual and household migration decisions. This is because the linkage of climate change to migration is not linear but dynamic and can be understood as an intrinsic part of broader social transformation processes (Castles et al., 2015). However, current thinking on climate-induced migration neglects the complexities of how migration alters the dynamics of societal transformations; and how the transformations in turn inform migration decisions.

We define social transformation as persistent changes in the cultural, economic, technological, and political structures at different levels of society (Castles, 2010). Complex interactions of climate-induced migration and social transformation complicate policy action. Social transformation processes, such as integrating migrants in receiving areas, and changing household roles in the sending areas, are critical issues requiring further unpacking for effective policy action. Also, there are many unanswered questions, such as how many people are expected to move, when, to where, and for what reason? What is the likely trend in the future, given climate change, growing conflicts, and the emergence of new employment opportunities? What future policy actions should be employed to support people who remain in their communities? Most studies on these issues focus on geographical regions within the USA, Mexico, and Bangladesh (Piguet et al., 2018), but insights from West Africa are generally lacking. There is, therefore, an urgent need to fill the gaps in knowledge, practice, and policy processes in West Africa. A better understanding of social transformation in sending and receiving areas will provide the information needed to design best-fit-for-purpose policies and interventions. The drivers and outcomes of social transformation processes will inform policymakers on climate risks and vulnerabilities and offer important lessons for building effective scenarios in policymaking and formulating interventions.

## 2 Methods

This study was grounded on a traditional literature review (Onwuegbuzie and Frels, 2016) and stakeholder dialogue (Conallin et al., 2017) in the form of a dialogue event to provide comprehensive, critical, and objective synthesis of the current knowledge on climate, migration, and social transformation. The information was collected between August 2021 and March 2022. Followed by a multistakeholder engagement. The review approach started with the sourcing of documents (articles and gray literature) using the Google Scholar search engine for the terms, “climate-induced migration,” “climate mobility,” “climate migration,” “climate related migration,” “climate migration and social transformation,” “climate-induced migration and West Africa,” and “social transformation.” This search engine was explored because, it provides many more articles, books and particularly gray literature that are generally difficult to get from other databases (Haddaway et al., 2015). The search was done for documents published in English and from 2000 upwards, to capture recent trends and discourse on the themes. It focused on the first 5 pages as relevant results, leading to an initial sample of 126 documents from a selection based on the review of titles and keywords. A quick review of the abstracts of the sample led to the inclusion of 38 key documents for full text review. The text information from the reviews of articles and policy documents, was extracted under the specific thematic areas with Atlas Ti version 8 and analyzed using an inductive qualitative content analysis (Graneheim and Lundman, 2004). The thematic content analyzed was synthesized based on a conceptualization of migration, social transformation, climate-related migration, climate situation in West Africa and its relation to migration, the gaps in policies on climate and migration, and the place of social transformation.

Insights were compiled into a brief, which was further summarized into a synopsis to inform discussions at a dialogue^1^ on “climate induced-migration and social transformation nexus: the policy issues.” The dialogue was attended by 50 participants from West Africa and beyond, including climate and migration experts from academia, policy and development planning, West African regional development organizations, the African Union, the Economic Community of West African States (ECOWAS), national and regional NGOs and civil society organizations, the European Union and United Nations, and the media. The dialogue provided an interactive platform to discuss what, how, where, and why climate intersects with social transformation factors to influence climate vulnerabilities and induce migration outcomes and the implications of these for climate, migration and gender policies and interventions? The insights from the participants were compiled and analyzed as information per the issues discussed. The synthesized information informed the framing of the gaps and questions for further reflections and research on climate-induced migration issues.

The findings of the review and information gathered from the dialogue are presented in the following sections, followed by reflections on how social transformation analysis can be used to better understand climate-induced migration in West Africa.

## 3 Conceptualizing climate-induced migration and social transformation

### 3.1 Climate change-driven migration

People migrating due to the impacts of climate change are usually described with words such as “climate migrants,” and “environmental migrants” (Dun and Gemenne, 2008). The International Organization for Migration (IOM, 2020a, p. 13) defines climate migration as “the movement of a person or groups who, predominantly for reasons of sudden or progressive change in the environment due to climate change, are obliged to leave their habitual place of residence, or choose to do so, either temporarily or permanently, within a state or across an international border.” The movement can be “voluntary or involuntary, triggered by a rapid-onset disaster or slow-onset climatic process, … informed by political factors and human intentionality” (McAdam, 2014, p. 17). Dehcheshmeh and Ghaedi (2020) observe that the movement can also be seasonal and singular. In recent times, the terminology “climate-induced migration” is increasingly being used in policy documents (Sharifi et al., 2021). This terminology, i.e., the movement of people driven by sudden or progressive changes in the weather or climate, is adopted in this paper.

There are two distinctive narratives on migration related to climate change. The maximalist (Morrissey, 2012) or alarmist (Myers, 1997; Bettini et al., 2017) narrative (migration due to failure to adapt in response to climate risks); and the minimalist narrative—migration as a form of risk reduction and responsive adaptive strategy (Kronlid and Grandin, 2014; Santos and Mourato, 2022). The latter views migration as a potential solution and is now highlighted in many policy initiatives. However, the former also persists, especially in media representations of climate-induced migration issues (Ransan-Cooper et al., 2015).

There are several theories, conceptual and analytical frameworks for unraveling migration issues. For instance, Mabogunje (1970) migration system theory explains why people would want to migrate and understand how the sending area is linked to a receiving area by the movement of people, flow of goods, capital, materials, ideas, and information in the system (Fussell et al., 2014). It highlights that a change in one part affects the entire system. Agrawal et al. (2008) and Bettini and Gioli (2016) attribute migration decisions to households based on their physical, natural, financial, human, and social assets. When affected by climate change, households diversify their risks by sending family members to less affected areas (Bettini and Gioli, 2016). The Roy-Borjas model (Roy, 1951; Borjas, 1987) focuses on how climatic events trigger liquidity constraints that inform the likelihood of emigration for the vulnerable. Others, like agent-based models (Smith, 2014), explore behavioral migration theory and inherent mobility potential to understand immobility under climate change by looking at the psychological propensity to migrate and residential satisfaction. Climatic impacts are non-linear and are context dependent (McLeman, 2018), and so are adaptive strategies, which also transform over time (Gemenne and Blocher, 2017). Existing frameworks do not capture these dynamics. Furthermore, the discourse around climate-induced migration has so far focused on the number of people involved, their characteristics, and water stress that can drive such migration (de Bruin et al., 2022; Färber et al., 2022).

This paper adopts the minimalist narrative by considering climate-induced migrant as an agent of climate adaptation (Methmann and Oels, 2015), i.e., a person who perceives migration as his or her best response to the impacts of climate change and acts on this perception. Several versions of this narrative are rooted in resilience building (Foresight, 2011). However, there is little direct linkage to a social transformation lens, despite evidence that climate-induced migration alters the social structure in sending and receiving areas (Castles, 2010; Williamson et al., 2021). The social transformation lens is needed to better understand climate vulnerabilities and migration decisions over time, and to support the implementation of transformative adaptation strategies in sending and receiving areas (Gavonel et al., 2021). Also, missing in the policy and programming frameworks is an in-depth analysis offering a clear understanding of how the changing climate impacts the dynamics, complexities, interconnectedness, variability, and multi-level mediations of economic and socio-cultural factors driving migratory decisions and processes. Understanding migration intentions and transformations will provide a basis for planning effective interventions (Alessandrini et al., 2020). However, the relationship between climate-induced migration and social transformation has not yet been clearly diagnosed, documented, and understood. Analyzing data on transformations in the economy, social networks and socio-culture at the household and community levels will reveal the contextual variabilities, complexities, and multi-level mediations of migration related to climate change. The analysis will unravel the challenges and opportunities, the drivers, the types, and processes of social transformation occurring, and the outcomes. These dynamics can be studied at different levels: the migrants themselves, the sending households and community and destination areas. The paper bridges the existing conceptual gap of climate-induced migration and social transformation.

### 3.2 Climate-induced migration and social transformation

Migration is a social process affected by social transformation processes (and vice versa) in both the origin and destination areas. Therefore, migration as a coping strategy to mitigate the impacts of climate change has implications for social transformation in both the sending and receiving areas. This section explains the interconnection between climate-induced migration and social transformation.

Social transformation is commonly referred to as an alteration in the social order of a society or a social structure. It refers to changes in culture, institutions, behaviors, relations, and structures over time. It can be assessed through different dimensions, including land size, technology, economy, inequality, and gender roles (Vago, 2004). The climatic (biophysical) factors influencing migration are changing, as are the social factors (e.g., poverty, gender roles, age, literacy rates), which exacerbate vulnerabilities to climate change (Osei-Amponsah and Quarmine, 2022). Social transformation can be seen both as a process (means) that drives, and as an outcome of, the effects of climate-induced migration.

Migration has been analyzed using theories based on the industrial revolution and other social, technological, demographic, and political events in the past (Singh and Basu, 2020). However, Castles (2010) argues that contemporary migration is part and parcel of the transformations brought about by globalization, which affects all forms of social interaction and all individuals and communities (except for the most remote and isolated) simultaneously. The concept—social transformation, therefore, serves as a lens for examining migration complexities, including that driven by the impacts of climate change. For instance, demographics, agricultural technologies, and farming practices are transforming (Lambrecht et al., 2018), which then influences migration decisions. Migration contributes to, and is affected by, rural and structural transformations (Paris et al., 2009). Using social transformation analysis as a lens can therefore provide insights into the implications for further transformations in gender roles, agricultural productivity, and wellbeing in rural West African households.

Climate-induced migration is dynamic, complex, and transformational (De Haas, 2010). It occurs in combination with other social trends. However, these are not captured in policies, or when they are, they are often framed as static and linear. In framing migration as an adaptive strategy, household assets (e.g., natural, financial, social) are substituted one for the other when needed to support livelihoods (Agrawal et al., 2008). Over time, based on available assets, households use risk diversification strategies as a basis for migration decisions, for example by sending member(s) to areas less affected by climate change to increase resiliency (de Sherbinin et al., 2012). The social and economic impacts vary based on the gender of the migrant, which in turn has implications for access to and control over productive assets and therefore influences the direction rural transformation takes (IFAD and FAO, 2008). Migration is increasing and is transforming communities. Transformations in family life, individual and group identities, intergroup relationships, and changing material landscapes of cities, industries and neighborhoods, influence migration decisions. For example, male migration tends to increase the workloads of females left behind, who must work longer hours on the farm and at the same time care for children and become household heads (Paris et al., 2009; Trisos et al., 2022). These women may not want to stay behind, but cultural norms may trap them in places with high climatic risk and vulnerability. On the other hand, male migration often leads to empowering women and provides them the freedom and autonomy to take on household management and community decision-making roles (Oucho and Ochieng, 2014). Furthermore, female migration contributes to strengthening the capacity of females, as they gain access to paid employment and a break from the often-rigid gender norms in rural communities (Chant, 2013). This situation notwithstanding, it is important to consider that, when females migrate, the males in addition to their normal productive tasks, must take on household roles like childcare duties, which are traditionally in the domain of females (Ramírez et al., 2005).

The flow of ideas, remittances, and social capital from migrants play a central role in transforming economic, social, and political life in places of origin and destination. When people migrate, they go through a process of self-transformation which in turn has an impact on the social, economic, and political structures (Adger et al., 2019). Migration can increase social inequality between migrant and non-migrant households in sending areas because of remittances received by migrant households. Migrants can improve their health, social and economic wellbeing in receiving areas, and contribute to the development of sending and receiving areas. Furthermore, migration of people to urban places can create urban slums, and sometimes little attention is paid to migrant integration into the urban system. The growing preference for nuclear families, coupled with migration, breaks social support systems down and leaves the aged without care in rural areas. Other sudden disruptions to development pathways (Osei-Amponsah and Wahabu, 2021), such as the COVID-19 pandemic, have affected migration trends and dynamics. Due to COVID-19 protocols and the associated economic crisis, migrants have been stranded and/or rendered jobless and unable to return to their rural communities. Those who returned suffer from the impacts of climate change such as drought, heat stress and food insecurity (Vinke et al., 2020).

The next section presents the contextual issues that highlights the climate change situation, governance and policy gaps of climate-induced migration.

## 4 The contextual issues

### 4.1 Climate change and migration in West Africa

The UN Framework Convention on Climate Change (UNFCCC), Article 1, defines climate change as: “a change of climate which is attributed directly or indirectly to human activity that alters the composition of the global atmosphere and which is in addition to natural climate variability observed over comparable time periods” (United Nations, 1992, p. 7). According to IPCC (2022, p. 1325) report, at 2°C global warming, West Africa is projected to experience a drier, more drought-prone and arid climate, especially in the last decades of the twenty-first century. Further, the duration of meteorological drought in the western parts of West Africa is projected to increase from approximately 2 months during 1950–2014 to approximately 4 months in the period 2,050–2,100 under RCP8.5 and SSP5-8.5 (Ukkola et al., 2020, cited in IPCC, 2022).

The impacts of extreme climatic events such as floods, drought, erosion, rising temperatures, and sea level rise are already significantly impacting people’s livelihoods. Some countries are concerned about desertification and drought; others are worried about hazards such as floods, coastal erosion, and sea level rise. For example, rainfall variability and drought lead to severe food insecurity, causing people to move in search of food. Similarly, sea-level rise combines with storms to create storm surges, leading to mass migration of people and livestock (Kirwin and Anderson, 2018). Climate-induced migration is projected to contribute significantly to the stock of global migrants, especially in regions that depend on natural resources, climate vulnerable activities (e.g., agriculture) and have weak capacity to adapt to climate change impacts. Agriculture in West Africa is largely rainfed and thus highly vulnerable to climate change. Reduced crop yields push youth out of agriculture (Shukla et al., 2021). Although promoting climate-smart agriculture is a positive response, the increasing impacts of climate change coupled with high population growth make it difficult to achieve adequate food and livelihood security.

The West African region has a long tradition of migration linked to kinship and religious networks, but climate change is contributing more and more to migration decisions; migration has become a key adaptation option for most rural households (UN DESA, 2020). The high rate of mobility may also, in part, be because of the ECOWAS Protocol on Free Movement of citizens of the 15 member-states. In many rural communities, both male and female youth migrate elsewhere within their country, to other West African nations, or even overseas to find alternative sources of livelihood (IOM, 2020b). Long distance moves are likely to sever local ties and undermine social support systems. There are also concerns about the effects of rapid urbanization driven by migration on the sustainability of cities. For example, city planners are confronted with the issues of sustainable urban growth, pressure on urban services and an increase in pollution and waste generation. Recently, the increasing migration of Fulani herders and other vulnerable populations from the Sahel and their settlement in countries along the coast has also become a source of concern for regional governments. This form of migration is triggered by climatic conditions and has also contributed to recurring conflict between herders and farmers or migrants and indigenous populations (Tonah, 2006; Bonye et al., 2021; Setrana and Kyei, 2021).

These conflicts have become significant security concerns. The flow of herders is mostly from the Sahel (Mali, Burkina Faso, Niger, and Chad), where dependence on a dry and deteriorating landscape is high, to countries where there are more plantations, mining, or other coastal activities, for example in Côte d’Ivoire, Ghana, Nigeria, Senegal, and The Gambia. Populations in the Sahel are finding it difficult to cope with the long periods of drought; traditional coping strategies are reaching their limits. For instance, the seasonal migration of young male adults from the savannah regions of Ghana into the transition zone to work as farm laborers has changed because of the impact of climate change on agriculture in the transition zone. These migrants now prefer to migrate to cities in the coastal and forest areas in search of other livelihoods (Rigaud et al., 2021).

### 4.2 Policies and programs on climate change and migration

Policymakers recognize climate change and migration as major development problems because of the vulnerable environmental conditions in the region. The Sustainable Development Goals (SDGs) do not specifically tackle climate change-related migration but addresses climate action (Goal 13). Increasingly, several overarching development agendas and policy strategies address climate-induced migration issues. For instance, UNFCCC (2010) acknowledges the importance of migration in the 2010 Cancun Adaptation framework; migration in the context of changing climate was also mentioned in ratification of the 2015 Paris Agreement (UNFCCC, 2015). Global institutions such as the UNFCCC, IOM, and the United Nations High Commissioner for Refugees (UNHCR) increasingly address the causes and consequences of climate migration as critical global priorities. Climate migration is also mentioned in the United Nations’ Global Compact for Safe, Orderly and Regular Migration (United Nations General Assembly, 2018), and the 2019 United Nations Human Rights Committee decisions (Le Moli, 2020).

The development framework for the African continent (AUC, 2015) identifies climate change adaptation as a critical issue. It does not directly refer to climate migration; however, the linkages between migration and security are mentioned. The AU Agenda 2063 is silent on climate-induced migration. For the ECOWAS region, there are instruments that align with climate-induced migration. Examples are the Free Movement of Persons, Residence and Establishment; Supplementary Protocol on Right of Residence and Supplementary Protocol on Right to Establishment. The Protocol on Free Movement allows nationals in member countries to travel to other ECOWAS countries for 90 days without a visa. It also allows for trade among member states, which facilitates migration in the sub-region. In addition, individual countries’ immigration policies recognize that migration has been used as an adaptation strategy to climate change and other environmental issues. These policies recognize the need to integrate migration and climate change into development and other sector plans in member states. Currently, most countries in the region have adopted institutional frameworks, laws, regulations, and action programs addressing pressing environmental issues, and climate change, such as the National Action Plans to Combat Desertification, the National Action Programs for Climate Change Adaptation, and Nationally Determined Contributions (NDCs). These programs highlight individual country’s priority areas and the resources required to implement actions identified in priority sectors but rarely address climate-induced migration.

The ECOWAS program for the sustainable management of pastoral resources and observation of transhumance is the most developed policy area directly concerned with climate-related and seasonal migration. ECOWAS is focused on promoting safe migration, combating illegal migration such as human trafficking, protecting of migrant’s rights, especially for refugees and asylum seekers, and harmonizing policies while paying attention to gender issues in migration. Regional dialogues such as the Migration Dialogue for West Africa and Euro-African Dialogue on Migration and Development (Rabat Process) seek to promote cooperation on policies and address migration issues.

The Sahel is high on the European Union’s foreign assistance agenda, focusing on development-related activities and the climate-migration nexus. EU’s development assistance to the region aims to address the root causes of migration and strategies to avoid displacement and irregular migration (European Union, 2011; Liguori, 2021). Other development partners fund and implement programs in the most affected sectors such as agriculture, health, energy, and water to ensure that people can cope with the challenges of climate change. For instance, U.S. foreign assistance addresses climate risk by managing droughts, promoting resilience to shocks and stresses, increasing water and food security, diversifying livelihoods, and improving access to affordable, quality health services through universal health care (White House, 2021). Others, including the International Social Science Council, International Development Research Center Canada, United Nations Development Program and Environmental Program, IOM, and UNHCR, are working on various climate change-related migration issues in the region.

The implementation of action plans in National Action Plans and NDCs by NGOs have helped member countries make headway in coping with the climatic situation. However, no specific national or regional policies directly address climate-induced migration. The next section discusses the reasons for gaps in the policies.

### 4.3 Policy gaps and areas of concern on climate-induced migration

Approaches to addressing climate-induced migration, which are also used in tackling broader migration issues in West Africa have been ineffective, largely due to inadequate knowledge and capacity to understand the dynamics as a basis for designing best-fit actions. Specific gaps curated from the stakeholder dialogue include:

- Inadequate data—due to the informal nature of the economies that send and receive migrants, official data in West Africa does not accurately identify and capture the number of migrants, and especially those displaced by climate change. Poor migration data hampers policymakers’ capacity to develop policies based on the characteristics of the people involved in migration.
- The links between climate change, migration and social transformation are not documented or mapped well enough to develop an adequate policy, research, and investment framework.
- Migration policies do not focus on urban dynamics (in addition to rural), particularly from social, economic, political, and environmental points of view.
- Agriculture is a key sector for intervention on migration and climate change. However, agricultural investments or migration policies rarely address its intersection with climate-induced migration.
- Migration is an important coping strategy for many people; however, it is currently not considered an entry point for building the region’s climate resilience.
- There is inadequate involvement of local actors in the formulation and implementation of strategies and interventions for managing climate-induced migration and its linkages.
- Gender is a key issue in different manifestations of migration, and while several gender analysis frameworks exist, they have not been adapted to the climate change-migration-social transformation nexus. Most important, gender transformative approaches are required for in-depth analysis of the issues.

## 5 An approach to social transformation analysis of climate-induced migration

### 5.1 The proposed conceptual framework

**Figure 1:**
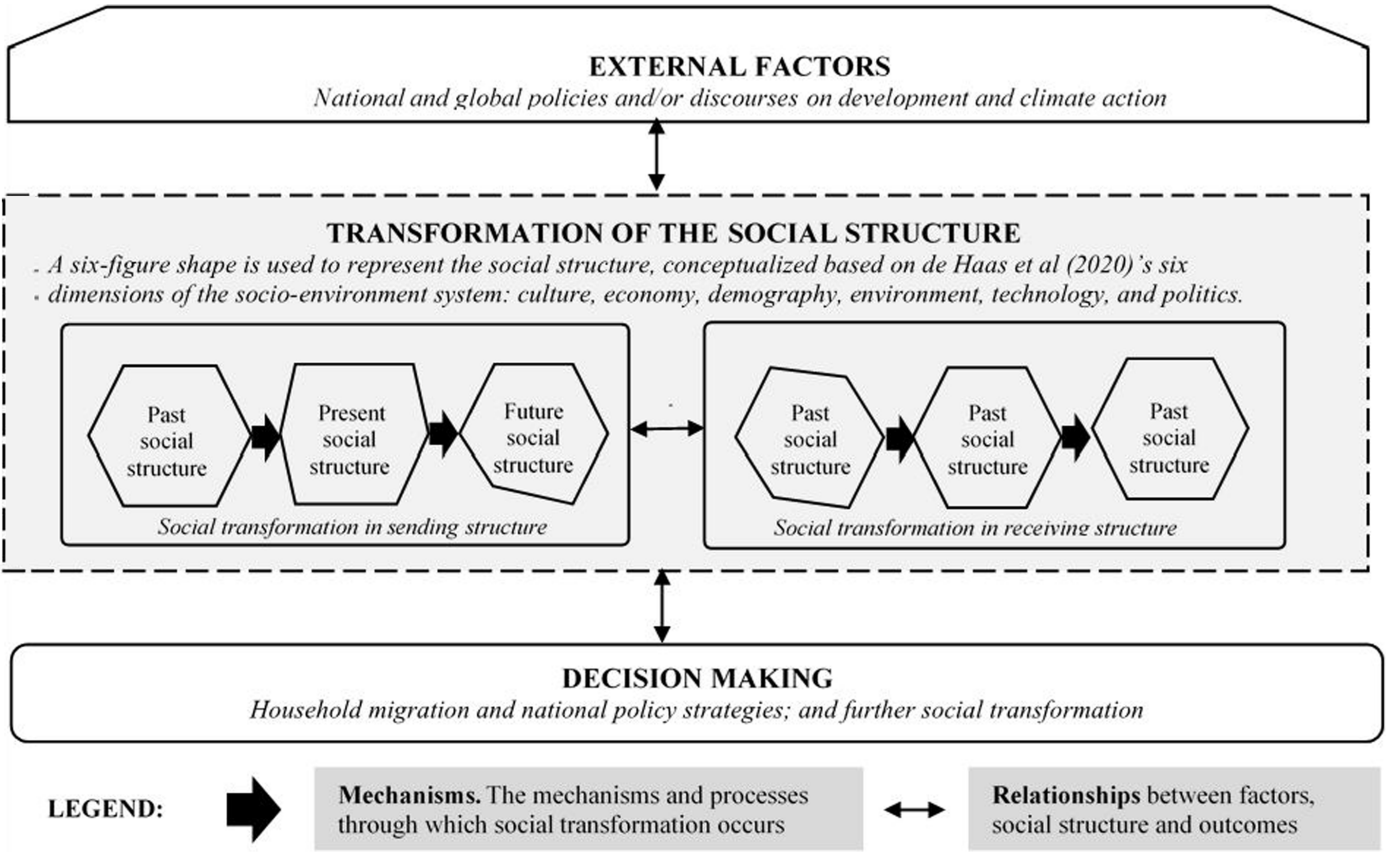
Proposed social transformation framework for understanding climate-induced migration.

A framework based on a sociological conceptualization of transformation is proposed as a lens through which researchers can unravel the complexity of climate-induced migration. The proposed framework (Figure 1) provides elements to analyze how social structures (community or household) transform, the nature of the transformations, the processes or mechanisms, typologies (extent and direction) of the outcomes, and implications of transformation for migration decision-making by different social groups.

The nature of the transformation is understood by unraveling what is persistently changing at multiple levels (household, community, national and global) because of multi-dimensional factors and their influence on climate change impacts and migration decisions over time. Here, information is gathered on the household, district, national, continental, and international transformational dynamics and interconnections. From a functionalist view, the process/mechanism of transformation is captured as: when an event (climate change) impacts/shocks a component (e.g., farming practice/system) of the social structure (household/community), creating disequilibrium and tension between that component and others, then all the components go through an adaptation/transformation phase to return to a (new) equilibrium (Bakamana and Kiingati, 2021). The information to be collected here also includes ways in which external and internal drivers interact with, and reinforce, multiple transformational outcomes.

The typologies relate to the outcomes of transformation. Information is captured on the internal structural transformation features at the household and community levels (e.g., changing gender norms, roles, and power relations), and the multiple values and meanings given by different social groups. At multiple levels, internal and external structural features of transformation (e.g., demographic trends, political economy, policies, governance systems, markets, development initiatives, ICT, innovations) should also be collected. The implications of transformation are assessed in terms of the decision-making processes of the climate-induced migrant, and the strategies and governance mechanisms for policymakers/development planners. The information will facilitate an understanding of how the different stakeholders can respond to the specific transformation typologies, to trigger further transformations to reinforce positive and minimize negative outcomes.

### 5.2 The research for development approach

To understand the dynamics and complexities of climate-induced migration, we propose social transformation as an analytical lens, using transdisciplinary research. This requires the engagement of diverse stakeholders (Ison, 2010) to identify the needs, interests, and values of all groups involved in transformation processes (UNESCO, 2006). Transdisciplinary research (Lang et al., 2012) involves different disciplines, problem definitions and solution pathways from diverse societal actors to effectively support robust climate resilience and migration solution pathways. The research entails working with social actors to identify contextual issues on the unit of analysis, climate change, resilience, migration; conduct detailed interdisciplinary research; implement capacity building and policy engagement activities; and evaluate and learn from actions toward transformative climate resilience and migration solutions.

Secondary data on contextual and political economy issues at different levels can be gathered through desk reviews. Primary data is collected mainly through ethnography (e.g., participant/non-participant observations, informal interactions, semi-structured interviews, and focus group discussions). In some cases, participatory rural appraisal methodologies may be employed (Chambers, 1997). Quantitative methods involving statistical analysis, econometrics, and institutional/policy analysis of existing large national datasets is required (Castles, 2010). This will provide a better understanding of demographic, socio-economic and political trends and structures driving the transformation processes.

The proposed framework enables collecting and analyzing data at multiple levels (e.g., sub-national, district/community and household levels), and integrating both quantitative and qualitative methods to comprehensively understand how social transformation influences climate-induced migration.

## 6 Entry points for consolidating climate-induced migration and social transformation thinking

Bridging the gaps in understanding the dynamics of climate-induced migration and policy effectiveness in West Africa calls for a holistic approach through interdisciplinary research and collaboration. Thus, we highlight potential entry points for consolidating climate-induced migration and social transformation thinking.

Sustainable climate research financing is critical to ensure that vulnerable communities and social groups are supported to prepare for, and adapt to, climate-related migration risks. It is, therefore, important to identify funding opportunities to support research designs and comprehensive analytical tools for a deeper exploration of nexus issues, inform climate-migration and social transformation assessments, and track and prioritize gaps.

Security is now an increasingly critical issue that must be integrated into development planning and migration policy. Climate change and growing social inequality are major drivers of conflict, which often exacerbate migration trends globally and in West Africa. Recognition of the complexity of the migration-climate change-social transformation-security nexus must be the starting point for research and policymaking and the pathways to manage, model, build scenarios, and forecast the complexity. Participatory scenario-building and modeling approaches can be employed to integrate climatic and dynamic socio-economic data. Researchers should not view the social structure as made up of a homogeneous group. Rather, specific needs of diverse social groups should be analyzed to support risk-informed decisions by households and proactive climate-migration adaptation planning by development officers.

Integrated cross-sectoral policy-making frameworks that effectively capture social transformation and climate-induced migration can support decision-making in various economic sectors (e.g., agriculture and natural resources). For example, in the 2030 SDG Agenda, article 29, migration is recognized as a “multidimensional reality” that is of “major relevance for the development of countries of origin, transit and destination” (United Nations, 2015). Although climate change and social transformation are also key components of the SDGs, there are no direct linkages between them. These issues require an integrated approach for effective policy making.

Climate-induced migration is increasing the number of female-headed households in West Africa as adult males migrate. In vulnerable environments, these households need to be identified and supported with alternative livelihood options. At the same time, “trapped population” is becoming a critical concern in the region; many households are trapped in vulnerable places because they do not have the means to migrate or due to cultural issues (e.g., females must stay behind while males migrate). Supporting the growing number of household members impacted by climate change who migrate, want to migrate but cannot, and should move but are not willing to do so, requires targeted policy measures. It is also critical to integrate social transformation analysis into broader development plans and the formulating and implementing of climate-induced migration strategies.

No single organization, institution, process, or partner can unpack the complexity alone. It will take collaboration and coordination among diverse actors/ stakeholders to analyze the issues and develop effective solutions. Effectively addressing the challenges of the climate change-migration nexus will require action from all stakeholders, including migrants, governments, international organizations, the private sector, and civil society actors. It is important to identify a core group of professionals and strategic stakeholders who can be used as an informal advisory group for brainstorming, networking, and keeping up with the latest developments in the nexus.

## 7 Conclusion

This paper presents the interconnections between climate-induced migration and social transformation in West Africa. An integrated social transformation analysis lens is suggested for ensuring that the complexities of climate-induced migration are effectively identified, and inclusive strategies planned, implemented, and sustained. It concludes that sustainable financing, transdisciplinary research approaches, and effective partnerships are critical to support the use of a social transformation lens for the planning and implementation of inclusive climate-induced migration policies.

## Author contributions

CO-A reviewed literature and drafted the abstract, introduction, methodology and findings. WQ and AO made equal contributions on conceptualizing the social transformation framework and conclusion section. WQ contributed to the visualization of the conceptual framework. The thinking through of the discussion, and coordination and revisions, was led by CO-A with support from WQ and AO. All authors contributed to the article and approved the submitted version.

## Footnotes

^1^The dialogue was organized by the International Water Management Institute under the Resilience Against Climate Change-Social Transformation Research (REACH-STR) project, in collaboration with the International Organization for Migration (IOM) and the Ghana Commission for United Nations Educational, Scientific and Cultural Organization, and supported by the Science and Technology Policy Research Institute of the Council for Scientific and Industrial Research, Centre for Migration Studies at the University of Ghana, and Simon Diedong Dombo University of Business and Integrated Development Studies.
